# Comparison of temporomandibular joint changes in Twin Block and Bionator appliance therapy: a magnetic resonance imaging study

**DOI:** 10.1186/s40510-014-0057-6

**Published:** 2014-10-01

**Authors:** Santosh J Chavan, Wasundhara A Bhad, Umal H Doshi

**Affiliations:** Department of Orthodontics and Dentofacial Orthopedics, Government Dental College and Hospital, Nagpur, Maharashtra 444003 India; Department of Orthodontics and Dentofacial Orthopedics, Saraswati-Dhanwantari Dental College and Hospital, Parbhani, Maharashtra 431401 India; UPHAR, 68, Builders Society, Near Nandanvan Colony, Aurangabad, Maharashtra 431002 India

**Keywords:** Temporomandibular joint, Twin Block, Bionator, Magnetic resonance imaging

## Abstract

**Background:**

The objective of the present study was to evaluate and compare temporomandibular joint changes especially disk-condyle-fossa relationship following functional treatment of skeletal class II division 1 malocclusion using Twin Block and Bionator appliances.

**Methods:**

The total sample consisted of 30 subjects (13 males and 17 females) with class II division 1 malocclusion having mandibular retrognathism, in the age group of 9 to 14 years. Two treatment groups, i.e., Twin Block and Bionator groups, were formed which comprised ten subjects each, while a group of ten subjects served as the control group. The treatment effects were evaluated using magnetic resonance imaging (MRI). For the treatment groups, pretreatment MRI with wax construction bite was taken. For all subjects, MRI images with corrected sagittal T1 images were recorded in a maximal intercuspation position at pretreatment (R1) and in an unstrained retruded position at the end of a 6-month observation period (R2).

**Results:**

At the end of 6 months of treatment, the condyles occupied a more anterior position in the fossa to its pretreatment position, while the disk moved more posteriorly in relation to the condyle. The control group showed no changes in the condyle and disk position over a period of 6 months.

**Conclusions:**

Although the treatment group showed consistent forward positioning of the condyle and backward movement of the disk, long-term MRI findings in these groups will further clarify the adaptations between the condyle fossa and articular disk.

## Background

Dentofacial deformities exist in the maxilla and/or mandible in all three dimensions of space but frequently occur in the anteroposterior plane manifesting as class II or class III malocclusion [1]. Out of these two, class II malocclusion is more common with a prevalence rate of 8.37% in Indian population [2].

Class II division 1 malocclusion can have discrepancies in all three dimensions in the form of narrow maxilla, high palate, and sagittal discrepancy. But the most consistent diagnostic finding in class II malocclusion is mandibular skeletal retrusion [3]. Hence, a therapy in the form of functional appliances that are able to enhance mandibular growth has been indicated in these patients [1,3]. The purpose of functional therapy is to change the functional environment of the dentition to promote normal function [1]. Most of the functional appliances are designed to enhance the forward growth of the mandible by encouraging a functional displacement of the mandibular condyles downward and forward in the glenoid fossa. This is balanced by an upward and backward pull in the muscles supporting the mandible. Adaptive remodeling may occur on both articular surfaces of the temporomandibular joint to improve the position of the mandible relative to the maxilla [1,4].

All the initial functional appliances evolved from the monobloc and later went through many modifications. Balters Bionator was one such modification [3,4]. But almost all these appliances share the limitation of being less patient friendly in terms of patients' ability to perform normal functions like eating and speaking. Hence, the goal of developing newer functional appliances such as the Twin Block appliance [1] was to produce a system that is simple, comfortable, and esthetically acceptable to the patient.

Numerous investigations have been carried out over the years to evaluate the possibilities of growth modifications with functional appliances; however, the results have not been equivocal. Some studies have reported significant effects while others have failed to demonstrate any consistent changes [5-7].

The effects of functional appliances on dentofacial structures have been sufficiently demonstrated by cephalometric studies [5-7]. However, we have limited knowledge of the changes in the temporomandibular joint. Concerns have been expressed regarding temporomandibular joint adaptation subsequent to functional appliance correction of class II division 1 malocclusion by anterior repositioning of the mandible [8]. Conventional imaging systems do not lend themselves to detailed study of the temporomandibular joint (TMJ) structures. Magnetic resonance imaging (MRI) offers a superior method for the evaluation of both the soft and hard tissues of the TMJ, but studies of functional appliance therapy using MRI are very limited [9-14]. This is especially true for the full-time-wear functional appliances like Twin Block and Bionator. So, the aims of the study were:

- To analyze and compare the temporomandibular joint changes in Twin Block and Bionator appliance therapy with controls by using MRI.
- To analyze and compare the position of the condyle in relation to the glenoid fossa and of the articular disk in relation to the condyle.

### Material

The total sample consisted of 30 subjects with class II division 1 malocclusion of which 13 were males and 17 were females in the age group of 9 to 14 years (Table 1). Informed consent form was signed by all subjects and their parents. Ethical committee approval was taken from the institute and university.

**Table 1:** **Sample description**

**Group**	**Number**	**Age (years)**	
		**Mean**	**SD**
Control			
Total	10	12	1.8
Male	3	11.9	1.9
Female	7	12.1	1.7
Twin Block			
Total	10	12.5	1.5
Male	6	12.6	1.6
Female	4	12.4	1.5
Bionator			
Total	10	11.5	1.6
Male	4	11.8	1.8
Female	6	11.3	1.4

All 30 subjects were divided into three groups in the form of control group, Twin Block appliance group, and Bionator appliance group with each group having ten patients (Table 1).

Both the appliances were of conventional type [1,4] with no modifications.

## Methods

For all the patients, routine diagnostic records like case history, clinical examination, study models, cephalogram, orthopantomogram (OPG), and photographs were taken. To study the temporomandibular joint changes, MRI was performed.

### Magnetic resonance imaging

MRI was performed at the Magnetic Resonance Imaging Center, using a 0.2-T Signa Profile MR System (GE Healthcare, Milwaukee, WI, USA) with bilateral TMJ coils of 9-in. diameter. Corrected sagittal T1 images were recorded in a maximal intercuspation position at pretreatment (R1) and in an unstrained retruded position at the end of a 6-month observation period (R2). An extra-pretreatment MRI was also obtained for the treatment group with the wax construction bite in position to assess the disk-condyle-fossa relationship at the postured position. To reduce the scanning time, the MRI was performed on the right TMJ only. The total scanning time required was approximately 15 to 20 min for each subject.

### Parameters used for magnetic resonance imaging

- Number of slices - 7
- Field of view (FoV) - 20 × 20 mm^2^
- Magnification - 2.0
- Matrix size - 190 × 160
- Number of excitation - 4
- Slice thickness - 3 mm
- Distance between slices - 1 mm

Measurements from the MRI included sagittal concentricity and sagittal disk position. Sagittal concentricity and sagittal disk position were measured from a T1-corrected sagittal image section through the center part of the condyle, which displayed the maximum length of the posterior border of the condyle and ramus.

### Sagittal concentricity

**Figure 1:**
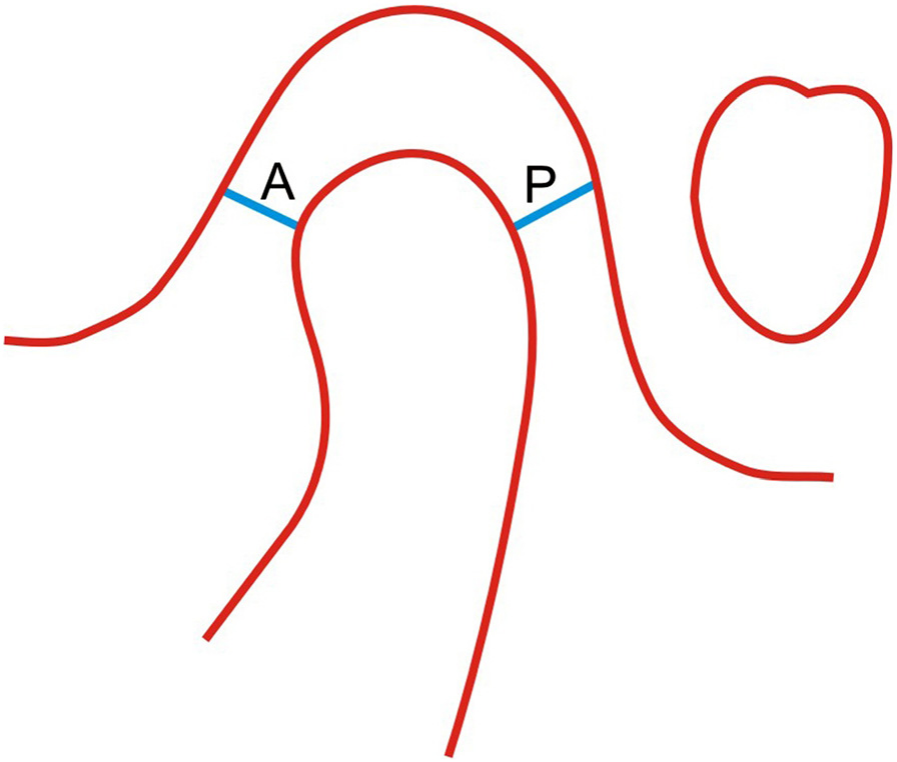
**Method of measuring sagittal concentricity.**

Sagittal concentricity (Figure 1) was evaluated using the method described by Pullinger et al. [15]. This denotes the position of the condyle within the joint in sagittal direction. It was calculated from the narrowest anterior and narrowest posterior interarticular joint spaces using the formula:

$$ \left[\left(P-A\right)\ /\ \left(P + A\right)\right] \times 100 = \%\ \mathrm{displacement} $$

Positive values indicated an anterior position, negative values indicated a posterior position, and a zero value was referred as to ‘concentric.’

The normal physiologic range of sagittal concentricity given by Vargas-Pereira [16] is 21.1 to −32.5.

#### Sagittal disk position

**Figure 2:**
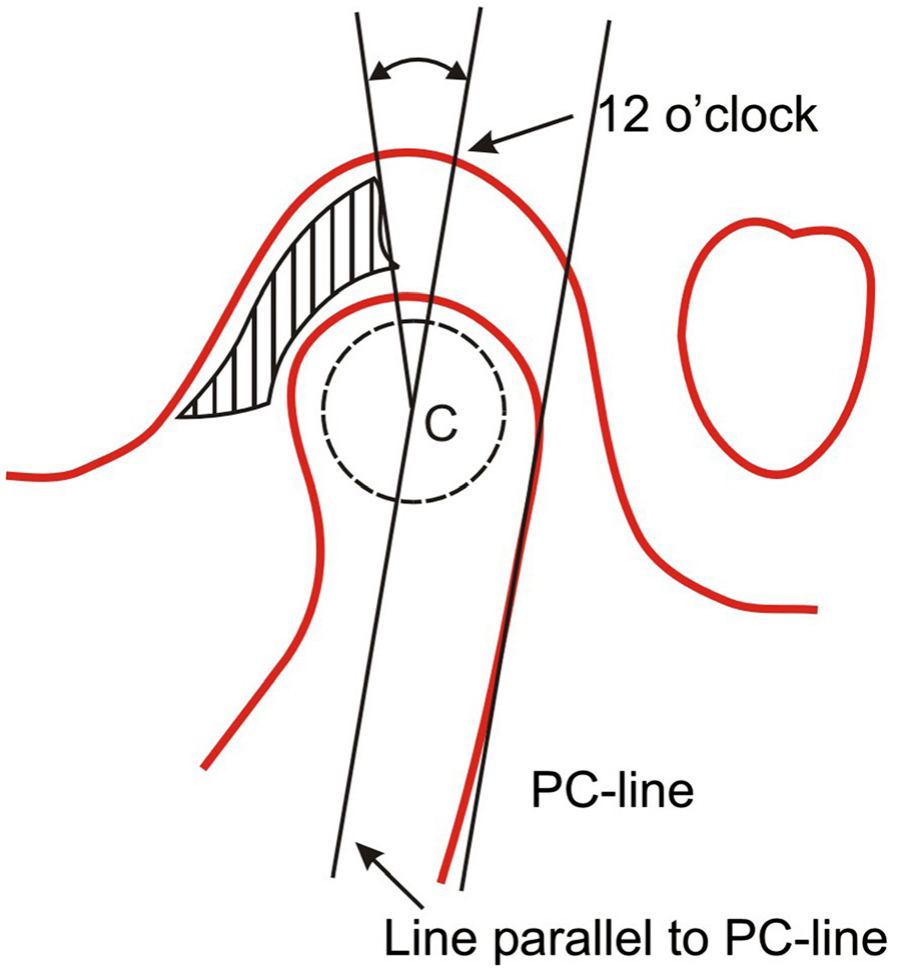
**Method of measuring sagittal disk position.**

The sagittal position of the articular disk (Figure 2) was assessed in the parasagittal MRIs of all patients involved in the present study by the method of defining disk position given by Chintakanon et al. [13]. This was a variation of the method used by Drace and Enzmann [17], who defined the so-called 12 o'clock position in determining disk position relative to the condylar head.

The intersecting point between a line parallel to the posterior condylar line passing through the condylar center and the roof of the fossa was constructed and referred to as the 12 o'clock position in the glenoid fossa. The position of the posterior bands of the disk was then measured as the angle relative to the 12 o'clock position. The position of the posterior band was used to classify the disk position into three categories: anterior displacement, normal, and posterior displacement.

The normal range for sagittal disk position given by Silverstein et al. [18] is 25.7° to −18.7° and Vargas-Pereira [16] is 33° to −21°.

The MRIs were interpreted visually by two different observers who underwent previous training to use the same protocol.

### Statistical method

All MRI parameters were measured. The data was tabulated and analyzed by SPSS© 8.0 software (SPSS Inc., Chicago, IL, USA).

Evaluations of intra-observer and inter-observer differences were performed in accord with Franco et al. [19]. A kappa of less than 0.4 was considered poor and a kappa greater than 0.75 was considered excellent.

Between-groups comparison of MRI variables was done by using unpaired *t* test. Paired *t* test was used to assess the difference in the rate of change of the different variables in the treatment group and control group. Significance was determined at the 0.05 and 0.01 levels of confidence.

- If *p* > 0.05, then it was not significant (NS)
- If *p* < 0.05, then it was significant (S)
- If *p* < 0.01, then it was highly significant (HS)

## Results

The assessment of intra-observer variability related to measurements of the sagittal concentricity yielded *k* = 0.5 for reading R1 vs R2. The evaluation of the sagittal disk position showed *k* = 0.7 for reading R1 vs R2.

Inter-observer kappa with regard to sagittal concentricity (*k* = 0.81) and position (*k* = 0.88) showed excellent agreement.

### MRI evaluation of sagittal concentricity

Comparison of mean values (Table 2) between the groups showed a highly significant difference between the control group and Twin Block group and the control group and Bionator group.

**Table 2:** **MRI evaluation: sagittal concentricity - comparison of mean values between the groups**

**Sagittal concentricity (percentage of condylar displacement)**	**Control group (C)** ***n*** **= 10**	**Twin Block (T)** ***n*** **= 10**	**Bionator (B)** ***n*** **= 10**	**Comparison**		
	***X*** **± SD (%)**	***X*** **± SD (%)**	***X*** **± SD (%)**	**C and T**	**C and B**	**T and B**
Pretreatment	6.9 ± 5.1	3.7 ± 2.3	7.7 ± 4.4	NS	NS	NS
After 6 months	6.7 ± 4.9	18.7 ± 10.3	19.1 ± 11.5	S	S	NS
Difference	−0.17 ± 0.2	15.01 ± 7.9	11.38 ± 7.1	HS	HS	NS

No significant difference was seen in the position of the condyle as measured by the sagittal concentricity at the start of the treatment for all the three groups.

After 6 months, a statistically significant change was seen in the condyle position between the control group and Twin Block group and the control group and Bionator group.

Comparison between the mean values before and after 6 months (Table 3) in each group showed a statistically highly significant difference for the Twin Block group and Bionator group, but no significant difference was seen in the control group.

**Table 3:** **MRI evaluation: sagittal concentricity - comparison between mean values before and after 6 months in each group**

**Group**	**Pretreatment (** ***n*** **= 10)**	**After 6 months (** ***n*** **= 10)**	***t*** **value**	**Result**
	**Mean ± SD (%)**	**Mean ± SD (%)**		
Control	6.9 ± 5.1	6.7 ± 4.9	−0.15	NS
Twin Block	3.7 ± 2.3	18.7 ± 10.3	4.42	HS
Bionator	7.7 ± 4.4	19.1 ± 11.5	5.60	HS

### MRI evaluation of sagittal disk position

Comparison of mean values (Table 4) between the groups showed a highly significant difference between the control group and Twin Block group and the control group and Bionator group. Pretreatment and after 6 months values of sagittal disk position between the three groups were not statistically significant.

**Table 4:** **MRI evaluation: sagittal disk position - comparison of mean values (degree) between the groups**

**Sagittal disk position**	**Control group (C)** ***n*** **= 10**	**Twin Block (T)** ***n*** **= 10**	**Bionator (B)** ***n*** **= 10**	**Comparison**		
	***X*** **± SD (deg)**	***X*** **± SD (deg)**	***X*** **± SD (deg)**	**C and T**	**C and B**	**T and B**
Pretreatment	11.2 ± 9.9	21.2 ± 9.3	15.5 ± 11.6	NS	NS	NS
After 6 months	8.6 ± 10.9	1.8 ± 0.2	−0.9 ± 0.5	NS	NS	NS
Difference	−2.6 ± 0.9	−19.4 ± 9.1	−16.4 ± 11	HS	HS	NS

Comparison between the mean values before and after 6 months (Table 5) showed no significant difference. Twin Block and Bionator groups showed a statistically highly significant difference in the sagittal disk position between the pretreatment and after 6 months values.

**Table 5:** **MRI evaluation: sagittal disk position - comparison between the mean values (degree) before and after 6 months**

**Group**	**Pretreatment (** ***n*** **= 10)**	**After 6 months (** ***n*** **= 10)**	***t*** **value**	**Result**
	**Mean ± SD (deg)**	**Mean ± SD (deg)**		
Control	11.2 ± 9.9	8.6 ± 10.9	−1.00	NS
Twin Block	21.2 ± 9.3	1.8 ± 0.2	−3.23	HS
Bionator	15.5 ± 11.6	−0.9 ± 0.5	−4.64	HS

## Discussion

The orthodontic literature is full of contradictory claims and differing results regarding the mandibular response to functional appliance treatment and the adaptability of the TMJ to this treatment. Some studies have reported radiographic changes in the human TMJ as a result of functional appliance therapy, but these studies have been limited to observation of bony changes in the sagittal view [6-8].

MRI has been the method of choice in recent years for simultaneous imaging of both the soft and hard tissues of the TMJ. The use of MRI to demonstrate TMJ adaptation following functional appliance (Herbst appliance) has been reported by Ruf and Pancherz [10]. The effects of Herbst and headgear-activator appliances have been studied, but comparisons between studies are complicated because of differences in MRI sequences and choice of reference landmarks. Moreover, these studies failed to distinguish between the effects of functional appliances and normal growth because comparisons with untreated controls were not done [13].

This study was designed to use MRI to examine, evaluate, and compare the changes in the condyle-disk-fossa assembly in class II division 1 cases, untreated (control) and treated with the Twin Block and Bionator appliances, over a period of 6 months, and to compare the difference in pretreatment and after 6 months values in all the three groups.

Two measurements were done on the sagittal MRIs:

- Sagittal concentricity
  Positive values of sagittal concentricity indicated an anterior position, and negative values indicated a posterior position. Zero referred to the concentric position of the condyle in the glenoid fossa.
  Pretreatment sagittal concentricity values of all the class II division 1 samples in the treated and untreated groups showed condyles distributed between anterior, concentric, and posterior positions but failed to demonstrate a significant difference in mean values. Though 60% of the condyles were located more anteriorly within the fossa, the values were within the physiologic range of 21.1 to −32.5 as given by Vargas-Pereira [16].
  This finding did not support the claim that mandibular retrognathic patients possess distally positioned condyles as a result of forward head posture. Ruf et al. [20] also found anteriorly positioned condyles in the majority of children studied and stated that this could be characteristic for class II division 1 malocclusion as has been reported earlier in both radiographs and MRI [12,13]. Arieta-Miranda et al. [21] have confirmed this finding using cone beam computed tomography in subjects with class II sagittal relation.
  In the present study, MRI with wax bite was taken before delivery of the appliances to visualize and confirm the anterior positioning of the condyle, which was nearly to the crest of the articular eminence in all the treated samples.
  Results after 6 months showed a significant difference in the position of the condyle in both Twin Block and Bionator groups demonstrating successful clinical findings. Comparison of sagittal concentricity between the pretreatment and after 6 months appliance-treated groups showed a highly significant difference. Anterior condylar position was seen in both Twin Block and Bionator groups compared to the pretreatment position as could be seen in MRI with wax bite in place.
  After treatment with the Twin Block appliance, it was interesting to observe that the condyles had apparently moved back and were repositioned in their glenoid fossa, while the occlusion of the treated children had changed from class II to class I. Although the condyles appeared to be seated in their fossae, the position of the condyle relative to the fossa was still anterior to its pretreatment position.
  Similar findings were reported by Chintakanon et al. [13] in their successfully treated Clark Twin Block group. Vargervik and Harvold [22] and Arat et al. [14] also made similar observations in activator-treated patients. Ruf and Pancherz [12] also reported anterior condylar position during Herbst treatment. However, they further reported that the condyle position reverted back as a result of settling of occlusion 1 year after the treatment period.
  Comparison of Twin Block and Bionator groups showed no significant difference after 6 months as it was obvious that the condyles were positioned anteriorly by both the appliances. Though the difference is non-significant, the Twin Block appliance showed more anterior positioning of the condyle.
  The control group showed a non-significant difference (0.15) between before and after 6 months values showing minimal change in condyle position.
- Disk position
  The sagittal position of the articular disk in relation to the condyle was assessed using the 12 o'clock criterion, a method given by Chintakanon et al. [13]. Disk position was considered normal if the thickest portion of the posterior band of the disk was situated between 11 and 1 o'clock positions [12]. Silverstein et al. [18] gave the normal range from 25.7° to −18.7° while according to Vargas-Pereira [16] the range was 33° to −21°. The disk with the thickest portion of the posterior band located anterior or posterior to this position was considered displaced. A positive value indicated an anterior disk position whereas a negative value indicated a posterior disk position.
  In the present study, pretreatment MRIs in all three groups showed two cases of anterior disk displacement (one Bionator, one Twin Block) whereas the remaining 28 were within the normal range. A slight tendency towards anterior disk displacement was noted by Ruf and Pancherz [12,23] more frequently in class II malocclusion.
  MRI with wax bite showed anterior displacement of the disk along with the condyle.
  Comparison between pretreatment and after 6 months disk position showed posterior movement from its initial pretreatment position in all treated cases but was within the physiologic range. Two cases with anterior disk displacement also showed a physiologic normal position after appliance therapy. These findings were in accordance with Ruf and Pancherz [12] and Pancherz et al. [11]. They found a slight retrusion of the disk after Herbst treatment. But studies by Arat et al. [14] showed no statistically significant changes in disk position, though the condyle was located anteriorly.
  For the control group, comparison of disk position before and after 6 months showed no statistical difference. Chintakanon et al. [13] in their study reported that the position of the disk appeared to move posteriorly and closer to 12 o'clock when comparing 6 months with initial records for both control and Twin Block.
  In the present study, when the change in the position of the disk was compared for Twin Block and Bionator groups after 6 months, the difference was non-significant with Twin Block showing more posterior positioning of the disk compared to Bionator.
  Thus, in our study, we found anterior positioning of the condyle relative to the pretreatment position but posterior to the initial registration position (wax bite).
  Sagittal disk position was more posterior in relation to the condyle at the end of 6 months of appliance therapy (Twin Block and Bionator).
  However, minimal change in the condyle-disk-fossa position was observed in the control sample of class II division 1 malocclusion over a period of 6 months.
  When Twin Block and Bionator were compared, Twin Block showed more anterior positioning of the condyle whereas the disk moved posteriorly. These findings are supported by Ruf and Pancherz [12].
  According to Ruf and Pancherz [12], the disk position changes seem to be the result of anterior condylar position immediately after treatment, which is known to be associated with a more posterior position of the disk relative to the condyle. However, they further reported that the disk position changes tended to revert during the posttreatment period from immediately after treatment to 1 year after treatment. To confirm these findings, further posttreatment longitudinal study is required.
  The findings from the present study are in contrast to those of Foucart et al. [9], who found that the mean position of the posterior band of the disk was located anteriorly after treatment with the Herbst appliance.

## Conclusions

MRI demonstrated translation of the mandibular condyle by the Twin Block and Bionator appliances to the crest of the eminence at the beginning of the treatment, but after 6 months of treatment, the mandibular condyle had apparently moved back into the glenoid fossa. However, the condyles occupied a more anterior position in the fossa, to its pretreatment position.

It appeared that the disk moved more posteriorly in relation to the condyle in the treatment group; however, this could be due to the condyle being moved more anteriorly by the appliance therapy.

The control group showed no change in the condyle and disk position over a period of 6 months. MRI findings revealed anterior positioning of the condyle in the fossa and posterior movement of the disk relative to the condyle in the treatment group.

However, further long-term MRI studies are required to assess the changes noted in the position of the condyle and disk in the fossa and their subsequent posttreatment adaptations with the use of Bionator and Twin Block appliances.
